# Dissecting Causal Relationships Between Gut Microbiota Imbalance, Inflammatory Cytokines, and Structural Connectivity in the Brain: A Mendelian Randomization Study

**DOI:** 10.1002/brb3.70980

**Published:** 2025-10-29

**Authors:** Qianling Guo, Dongli Yang, Aamir Fahira, Qiusheng Zhong, Jiahao Yang, Kai Zhuang, Ying Wen, Zhuolun Tang, Zunnan Huang

**Affiliations:** ^1^ The Affiliated Dongguan Songshan Lake Central Hospital Guangdong Medical University Dongguan Guangdong China; ^2^ Guangdong Medical University Key Laboratory of Big Data Mining and Precision Drug Design, Guangdong Provincial Key Laboratory For Research and Development of Natural Drugs, School of Pharmacy Guangdong Medical University Dongguan Guangdong China; ^3^ School of Biomedical Engineering Guangdong Medical University Dongguan Guangdong China; ^4^ The Second School of Clinical Medicine Guangdong Medical University Dongguan Guangdong China

**Keywords:** brain structural connectivity, gut microbiota, magnetic resonance imaging, mendelian randomization, microbiota‐gut‐brain axis

## Abstract

**Purpose:**

Growing evidence indicates that the imbalances in gut microbiota influence brain structural connectivity, a key component of the microbiota‐gut‐brain axis. However, a deeper understanding of this complex bidirectional relationship remains elusive. This study aims to deepen our understanding of this bidirectional relationship by examining the underlying causal relationship and the mediating role of inflammatory cytokines.

**Methods:**

This study analyzed GWAS data from 18,340 participants to investigate gut microbiota composition and MRI data from 82,382 participants to examine brain structural connectivity. We conducted a bidirectional two‐sample Mendelian randomization (MR) to explore potential causal relationships between 211 gut microbiota taxa and 206 brain connectivity features. A two‐step mediation analysis involving 41 inflammatory cytokines was performed, using the inverse variance weighted (IVW) method as the main analytical approach, supplemented by sensitivity analyses and reverse MR to check for robustness, reverse causation, heterogeneity, and horizontal pleiotropy.

**Finding:**

After Bonferroni correction, MR analysis identified significant correlations between 11 pairs of gut microbiota taxa and brain connectivity traits, with six positive and five negative associations. Reverse MR confirmed positive associations in nine pairs. Sensitivity analyses found no evidence of horizontal pleiotropy, heterogeneity, or reverse causality. Inflammatory cytokines, such as RANTES, HGF, and IL‐13, mediated 10%–30% of these relationships, mainly through JAK‐STAT, IL‐17, and MAPK pathways.

**Conclusion:**

This research establishes potential causal links between gut microbiota and brain structural connectivity, bridging a crucial gap in the microbiota‐gut‐brain axis research. These findings enhance our understanding of the axis and suggest new therapeutic targets for neurological disorders.

## Graphical abstract

This study identifies causal links between gut microbiota and brain structural connectivity using Mendelian randomization and mediation analyses. Inflammatory cytokines, including RANTES, HGF, and IL‐13, mediate these effects through immune pathways, revealing novel insights into the microbiota‐gut‐brain axis and potential therapeutic targets for neurological disorders.

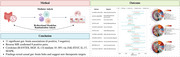

**Abbreviations**
AD: Alzheimer's disease
ADHD: Attention deficit hyperactivity disorder
B‐NGF: Nerve Growth Factor‐β
CI: Confidence intervals
FDR: False discovery rate
GMV: Gray matter volume
GO: Gene Ontology
GWAS: Genome‐wide association studies
HGF: Hepatocyte growth factor
IBD: Inflammatory bowel disease
IL‐12_P70: Interleukin‐12
IL‐13: Interleukin‐13
IL‐17: Interleukin‐17
IL‐18: Interleukin‐18
IL‐1RA: Interleukin‐1 receptor antagonist
IL‐2: Interleukin‐2
IL‐4: Interleukin‐4
IL‐6: interleukin‐6
IL‐7: Interleukin‐7
IVs: Instrumental variables
IVW: Inverse variance weighted
JAK‐STAT: Janus Kinase‐signal transducer and activator of transcription
KEGG: Kyoto encyclopedia of genes and genomes
LD: Linkage disequilibrium
LH‐DAN to RH‐DAN: Left‐hemisphere dorsal attention network to right‐hemisphere dorsal attention network white‐matter structural connectivity
LH‐DAN to RH‐LN: Left‐hemisphere dorsal attention network to right‐hemisphere limbic network white‐matter structural connectivity
LH‐LN to LH‐CN: Left‐hemisphere limbic network to left‐hemisphere control network white‐matter structural connectivity
LH‐LN to RH‐DMN: Left‐hemisphere limbic network to right‐hemisphere default mode network white‐matter structural connectivity
LH‐S/VANN to RH‐CN: Left‐hemisphere salience/ventral attention network to right‐hemisphere control network white‐matter structural connectivity
LH‐SMN to RH‐DAN: Left‐hemisphere somatomotor network to right‐hemisphere dorsal attention network white‐matter structural connectivity
LH‐SMN to RH‐SMN: Left‐hemisphere somatomotor network to right‐hemisphere somatomotor network white‐matter structural connectivity
LH‐VN to HC: Left‐hemisphere visual network to hippocampus white‐matter structural connectivity
MAPK: Mitogen‐activated protein kinase
mbQTL: Microbial quantitative trait loci
MCP‐1/MCAF: Monocyte chemotactic protein 1/monocyte chemotactic and activating factor
MCP‐3: Monocyte chemotactic protein‐3
M‐CSF: Macrophage colony‐stimulating factor
MIG: Membrane immunoglobulin
MIP‐1B: Macrophage inflammatory protein‐1‐β
MR: Mendelian randomization
MRI: Magnetic resonance imaging
MR‐PRESSO: Mendelian randomization Pleiotropy RESidual Sum and Outlier
OR: Odds ratio
PD: Parkinson's disease
PDGF‐BB: Platelet‐derived growth factor‐BB
RANTES: Regulated upon activation normal T cell expressed and secreted
RH‐SMN to CA: Right‐hemisphere somatomotor network to caudate white‐matter structural connectivity.
rRNA: Ribosomal RNA
SCFAs: Short‐chain fatty acids
SNPs: Single nucleotide polymorphisms:
TNF‐B: Tumor necrosis factor‐β
TNF‐α: Tumor necrosis factor‐alpha
TRAIL: TNF‐related apoptosis‐inducing ligand
UVMR: Univariable mendelian randomization
VEGF: Vascular endothelial growth factor

## 1 Introduction

The gut microbiota, a complex microbial ecosystem, influences brain development and behavioral performance through various mechanisms. This microbial community communicates extensively with the central nervous system via the microbiota‐gut‐brain axis, which involves neural, endocrine, and immune pathways (Cryan et al. 2019). Studies have indicated that specific microbiota can indirectly influence brain structure and function by affecting the barrier function of the gut epithelium and producing metabolic products such as short‐chain fatty acids (SCFAs) and bile acids (Caspani and Swann 2019). Observational studies have previously identified an association between gut microbiota imbalance and structural changes in the brain related to various neurodegenerative and psychiatric disorders, including Alzheimer's disease (AD) (Sochocka et al. 2019), Parkinson's disease (PD) (Sampson et al. 2016), autism spectrum disorder (Fetissov et al. 2019), epilepsy (Dahlin and Prast‐Nielsen 2019), and major depressive disorder (Zheng et al. 2016). Concisely, patients with Alzheimer's disease often exhibit significant differences in gut microbiota (Li et al. 2024a), accompanied by structural changes such as hippocampal atrophy (Zhao et al. 2017). Despite these observational studies providing preliminary evidence of gut‐brain interactions, they are often confounded by various factors, making it challenging to establish causal relationships. Therefore, further investigation is needed to elucidate the causal relationship between gut microbiota and changes in brain structure.

Inflammation plays a pivotal role in shaping both brain structure and function, with inflammatory cytokines such as tumor necrosis factor‐alpha (TNF‐α) and interleukin‐6 (IL‐6) serving as key mediators (Bagyinszky et al. 2017; Smith et al. 2012). Inflammation is implicated in structural brain changes underlying neuropsychiatric disorders via microglia and astrocytic function, leading to disordered synaptic pruning and the subsequent effects on gray matter volume (GMV) (Khandaker et al. 2020). The gut microbiota and its metabolites can regulate the function of local immune cells in the brain, thereby influencing neural responses and altering brain structure. Briefly, SCFAs can modulate cytokine production to regulate blood‐brain barrier permeability and modulate microglial activity, playing a critical role in maintaining brain health (Agirman et al. 2021). Recent Mendelian randomization (MR) analyses have provided evidence suggesting a potential causal relationship between inflammation and changes in brain structures (Williams et al. 2022). The intricate interplay between the gut microbiota and brain structural connectivity, mediated by inflammation, is crucial for advancing our understanding of immunomodulatory mechanisms in gastrointestinal and neurological disorders. However, the specific role of the gut microbiota in modulating brain structure through inflammatory mediators remains unexplored. This study aims to address this gap by investigating these relationships through Mendelian randomization and mediation analysis.

MR has emerged as a powerful tool for uncovering potential biological causal relationships. This technique relies on genetic variation as a naturally randomized instrumental variable, using the correlation of these variations with exposure to assess potential causal effects (Davey Smith and Hemani 2014). Recently, MR has been widely applied to investigate the causal relationship between gut microbiota and various diseases. Concisely, this method has been used to study the relationship between gut microbiota and neurological diseases, with results supporting the notion that gut microbiota imbalance may increase the risk of depression (Qi et al. 2021). However, despite extensive research on the causal links between gut microbiota and systemic health conditions, no studies have yet explored the direct causal relationship between gut microbiota and brain structural connectivity using MR. Given the complexity of the microbiota‐gut‐brain axis and its potential role in neurodegenerative and mental health diseases, investigating how gut microbiota influences brain structural connectivity will fill an important knowledge gap and may provide a foundation for developing new therapeutic strategies.

This study investigates the causal relationship between gut microbiota and brain structural connectivity using a two‐sample Mendelian randomization approach. By elucidating these causal links, the findings contribute to a deeper understanding of the microbiota‐gut‐brain axis and its role in brain health. Moreover, this research may pave the way for novel diagnostic and therapeutic strategies targeting the gut microbiota, offering potential interventions for neurodegenerative diseases and other related neurological disorders.

## 2 Materials and Methods

### 2.1 Study Design

**FIGURE 1:**
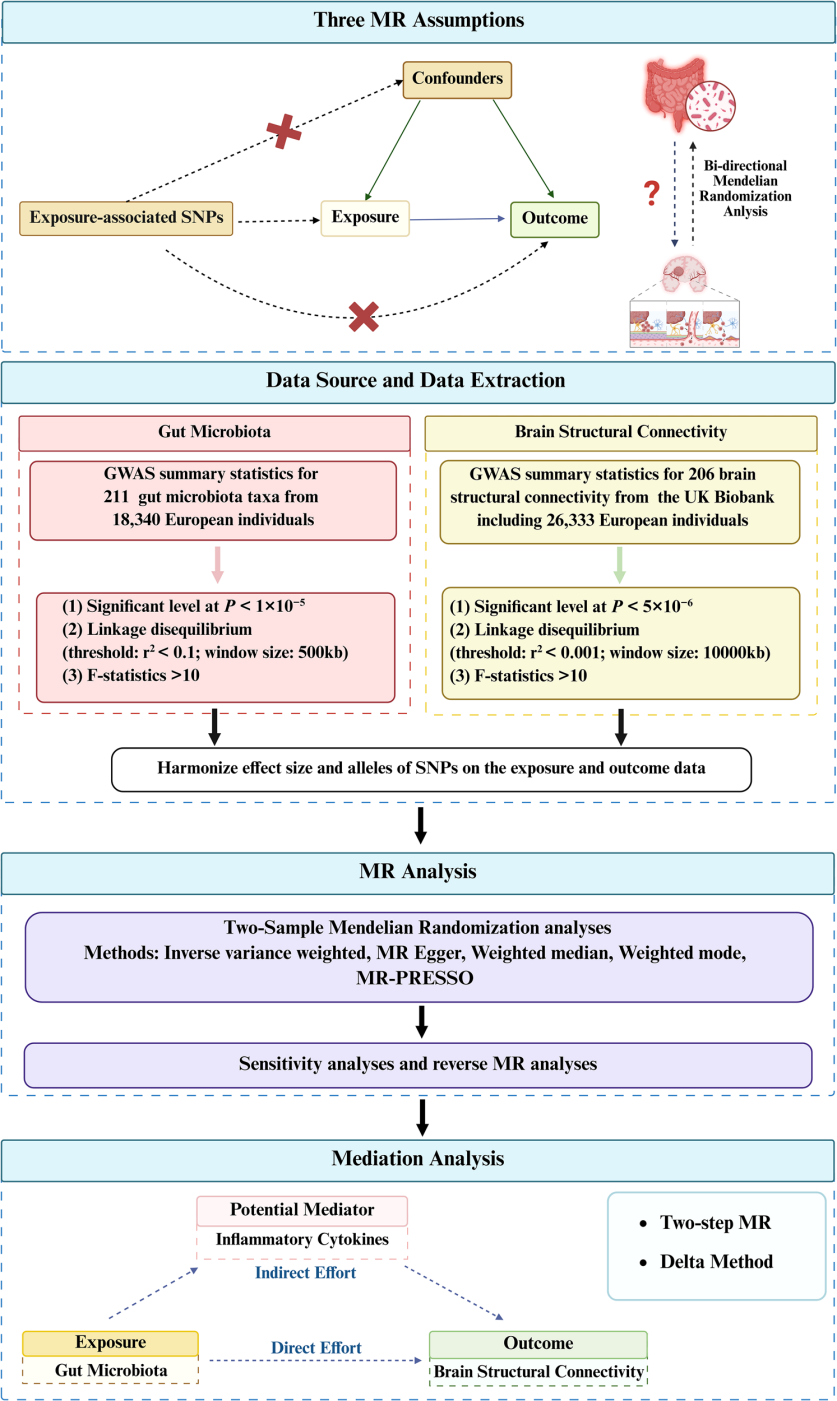
A Schematic diagram of MR Analysis. ***Note***: GWAS: Genome‐wide association studies; SNP: single‐nucleotide polymorphisms. (Figure created with biorender.com).

The flowchart of the study is shown in Figure 1. The data analyzed in this study were obtained from publicly available, previously published genome‐wide association studies accessible through the GWAS Catalog (https://www.ebi.ac.uk/gwas/). Therefore, all original research was ethically approved, and informed consent was obtained. The study utilized 206 brain structural connectivity phenotypes and 211 taxonomic units of aggregated gut microbiome data from GWAS. Before MR analysis, the instrumental variables (IVs) were rigorously screened. Three core assumptions must be met for MR which comprise (i) The assumption of association, that is, the IVs must be strongly associated with the exposure, as determined by GWAS; (ii) The assumption of independence, that is, the IVs are not associated with any confounding factors influencing the exposure and the outcome; and (iii) The assumption of exclusion restriction, that is, the IVs influence the outcome variable only through the exposure and not through other pathways (Davey Smith and Hemani 2014). We then performed bidirectional two‐sample MR analyses as well as sensitivity analyses. Furthermore, a two‐step Mendelian Randomization approach was employed to screen for potential mediators among 41 inflammatory cytokines and to quantify their mediating effects in the causal associations between gut microbiota and brain structural connectivity. These comprehensive analyses not only ensure the validity of the causal inferences drawn from the MR approach but also provide a deeper understanding of the underlying mechanisms, particularly by identifying potential mediators and quantifying their effects. This thorough investigation enhances the reliability of the findings and offers new insights into the causal pathways linking gut microbiota and brain structural connectivity.

### 2.2 Data Sources for Gut Microbiota

Single‐nucleotide polymorphisms (SNPs) associated with the composition of the human gut microbiome were selected as IVs from the GWAS dataset of the MiBioGen international consortium (https://mibiogen.gcc.rug.nl/). This dataset encompasses 18,340 European participants from 24 independent cohorts. Microbial composition was analyzed by targeting three different variable regions of the 16S rRNA gene, which resulted in an estimated 5,717,754 SNPs. Two hundred and eleven taxa (9 phyla, 16 classes, 20 orders, 35 families, and 131 genera) that fit the mbQTL (microbial quantitative trait loci) mapping analysis were included in this study (Kurilshikov et al. 2021).

### 2.3 Data Sources for Brain Structural Connectivity Features

This study utilized structural brain connectivity data sourced from the United Kingdom Biobank, encompassing 26,333 individuals of European genetic ancestry (Wainberg et al. 2024). Briefly, Wainberg et al. (2024) conducted magnetic resonance imaging (MRI) scans, incorporating both T1‐weighted and diffusion‐weighted sequences. T1 scans were employed for structural imaging, providing surface model files and additional structural segmentation. Conversely, diffusion‐weighted MRI scans were utilized to capture white matter structural connections. Furthermore, quality control metrics such as signal‐to‐noise ratio, contrast‐to‐noise ratio, and assessment of head motion were employed to ensure data reliability. Only scans that met these quality control criteria were included in subsequent analyses, thereby ensuring the integrity of the imaging data used to characterize brain connectivity. The dataset encompasses three categories of structural brain connectivity measures, totaling 206 in all. These include hemisphere‐level cortical‐to‐cortical connectivity (3 measures), network‐level cortical‐to‐cortical connectivity within and between each of the 14 hemisphere‐specific “Yeo 7” (Yeo et al. 2011) networks (105 measures), and cortical‐to‐subcortical connectivity between each “Yeo 7” network and 7 subcortical structures (98 measures) (Wainberg et al. 2024). Statistical data are publicly available in the GWAS catalog under accession numbers GCST90302648 through GCST90302853.

### 2.4 Data Sources for Inflammatory Cytokines

The GWAS conducted by Ahola‐Olli et al. (2017) provided data on circulating cytokines and growth factors. This study uses a dataset containing the genome‐wide meta‐analysis summary statistics data of 41 inflammatory cytokines, conducted within three Finnish cohorts (YFS and FINRISK 1997 and 2002), encompassing 8,293 individuals with European ancestry (Corbin and Timpson 2020). The dataset provides comprehensive genetic mapping of cytokines implicated in inflammatory processes, offering valuable insights into their regulatory mechanisms. This dataset was used in mediation analyses to explore the relationships between gut microbiota, inflammatory cytokines, and brain structural connectivity, aiming to uncover the pathways linking the gut microbiota to changes in brain structure.

### 2.5 Selection of Instrumental Variables

To delve into the relationship between the gut microbiome and brain structural connectivity, we applied a significance threshold of *p* < 1 × 10^−5^. While this threshold is more lenient than the conventional genome‐wide significance level (*p* < 5 × 10^−8^), it was chosen to ensure an adequate number of instrumental variables (IVs) for analysis. Given the highly polygenic nature of gut microbiota, adopting a stricter threshold would have resulted in too few IVs, thereby reducing the statistical power of our MR analysis. This relaxed *p*‐value threshold is widely adopted in microbiome‐related MR studies to balance statistical power and instrument validity (Li et al. 2024b; Li et al. 2022a; Qin et al. 2024).

In addition, for brain structural connectivity and inflammatory cytokines, we used SNPs with a significance threshold of *p* < 5 × 10^−6^ (Zheng et al. 2024) as genetic tools.

To ensure the selection of an independent SNP locus, we performed linkage disequilibrium (LD) analysis using the “clump_data” function of the R package “TwoSampleMR” (Hemani et al. 2018). The screening criteria for the gut microbiota were set at *r* ^2^ = 0.1 and kb = 500; SNPs with *r* ^2^ greater than 0.1 to the most significant SNP in the range of 500 kb were excluded (Hemani et al. 2018). For brain structural connectivity and inflammatory cytokines, the screening criteria were set at *r* ^2^ = 0.01 and kb = 10,000. The R package "TwoSampleMR” was used to analyze the association between the exposure factors and the outcome phenotypes. Consistent analysis of the effect alleles of SNPs associated with both exposure and outcome phenotypes was conducted to ensure consistent effect alleles and to exclude SNPs with palindromic structures. Furthermore, to assess the strength of selected SNPs, the following formula (Levin et al. 2020; Palmer et al. 2012) was used to compute the *R* ^2^ and F‐statistic corresponding to each SNP, and SNPs with an F‐statistic less than 10 were excluded to avoid the introduction of a weak instrumental variable bias (Table S1–S3).

$$F=\frac{R^{2}\times \left(N\;-2\right)}{1-R^{2}}$$

*R* ^2^ denotes the IV explanation of exposure, also known as PVE (phenotypic variance explained), and *N* denotes the sample size.

### 2.6 MR Analysis

The primary analysis of this study uses the inverse‐variance weighted method (IVW) (Burgess et al. 2015) to assess the causal relationship between gut microbiota and brain structural connectivity. For each association, the odds ratio (OR) and 95% confidence intervals (CI) were then calculated. Specifically, effect sizes and standard errors for both exposure and outcome were obtained for each genetic variant. A weighted sum of the effects, represented by the genetic instruments, is computed to determine the overall effect size. In addition, multiple tests were conducted, such as MR‐PRESSO (Verbanck et al. 2018), weighted median (Bowden et al. 2016), weighted mode (Hartwig et al. 2017), and MR‐Egger (Burgess and Thompson 2017). Moreover, a bi‐directional MR analysis (Hemani et al. 2018) was conducted to investigate the presence of reverse causal relationships.

### 2.7 Mediation MR Analysis

A two‐step MR analysis (Relton and Davey Smith 2012) was performed to explore the potential mediating role of inflammatory cytokines in the association between the gut microbiota and brain structural connectivity. In the first step, univariable MR (UVMR) was employed to assess the causal effect of the genetically determined gut microbiota and inflammatory cytokines (*β*1). The second step involved estimating the causal impact of each inflammatory cytokine as a mediator on brain structural connectivity (*β*2), assuming that the mediator is causally linked to the UVMR outcome. The mediation proportion of each mediator in the association between the gut microbiota and brain structural connectivity was calculated by the following formula (VanderWeele 2016):

$$\;MP_{n}=\frac{\beta_{n1}\;\times \;\beta_{n2}}{\beta_{\mathrm{ntotal}}}$$

where *β*_n1_ represents the causal effect for each gut microbiota‐inflammatory cytokine pair, *β*_n2_ represents the causal effect for each inflammatory cytokine/brain structural connectivity, *β*_ntotal_ represents the total causal effect for each gut microbiota/brain structural connectivity pair, and MP_n_ represents the mediation proportion for each pair (Vanderweele 2015). The mediated proportion represents the percentage of the total causal effect of a gut microbiota on brain structural connectivity that is indirectly transmitted through a specific inflammatory cytokine. Confidence intervals were estimated using the delta method (Kendall et al. 1994).

### 2.8 Enrichment Analysis

Functional enrichment analysis of 41 cell cycle factors was performed using the Metascape database (https://metascape.org/), focusing on Gene Ontology (GO), KEGG, and Reactome pathways. To visualize the results, a circular plot was employed, effectively highlighting the enriched terms. Significant terms were further subjected to hierarchical clustering based on Kappa‐statistical similarities (Cohen 1960) among their associated gene sets. Using a Kappa score threshold of 0.3, the hierarchical tree was partitioned into distinct term clusters, facilitating the identification of functional modules. The *p*‐value cutoff was set at 0.01 to ensure statistical significance, and the minimum enrichment threshold was defined as 1 to include all relevant terms.

### 2.9 MR Sensitivity Analysis

To assess the robustness of the results, a series of sensitivity analyses were conducted. Concisely, heterogeneity was evaluated using Cochran's Q test (Greco et al. 2015), with a *p‐value* < 0.05 indicating the presence of heterogeneity. MR pleiotropy residual sum and outlier (MR‐PRESSO) (Verbanck et al. 2018) were performed to further explore the stability of the results. When the global test *p*‐values in the MR‐PRESSO analysis were less than 0.05, the estimates were adjusted for outliers. The MR‐Egger intercept test and MR‐PRESSO global test were utilized to detect the influence of pleiotropy on causal association estimates, with a *p‐value* < 0.05 indicating the presence of horizontal pleiotropy (Bowden et al. 2015). The reliability of the association was assessed through leave‐one‐out analysis, funnel plots, and scatter plots. Briefly, a leave‐one‐out analysis (Burgess et al. 2017) was performed to ensure that no bias was caused by a specific SNP. Scatter plots demonstrate that the results are not influenced by outliers. Funnel plots (Burgess et al. 2017) are utilized to evaluate the reliability of the association. Furthermore, to minimize the risk of false positives, the Bonferroni correction was applied. This adjustment accounted for the number of bacterial taxa in the gut microbiome, setting a more stringent significance threshold: 2.37 × 10^−4^ (0.05/211) for forward analysis and gut microbiota to mediation in mediation analysis. Reverse causality analyses were then performed to investigate whether brain structural connectivity could influence the gut microbiota, with a Bonferroni correction of 2.43 × 10^−4^ (0.05/206) applied for reverse analysis. Additionally, MR analysis was conducted between mediation and brain structural connectivity, with a Bonferroni‐corrected significance threshold of 0.0012 (0.05/41).

### 2.10 Statistical Analysis

All analyses were performed in R software version 4.3.1 (https://www.r‐project.org/). The IVW, weighted median, MR‐PRESSO, MR‐Egger, and sensitivity analyses were conducted using the TwoSampleMR package (version 0.5.7) and the MR‐PRESSO package (version 1.0).

## 3 Result

### 3.1 The Causal Effect of Gut Microbiota on Brain Structural Connectivity

**FIGURE 2:**
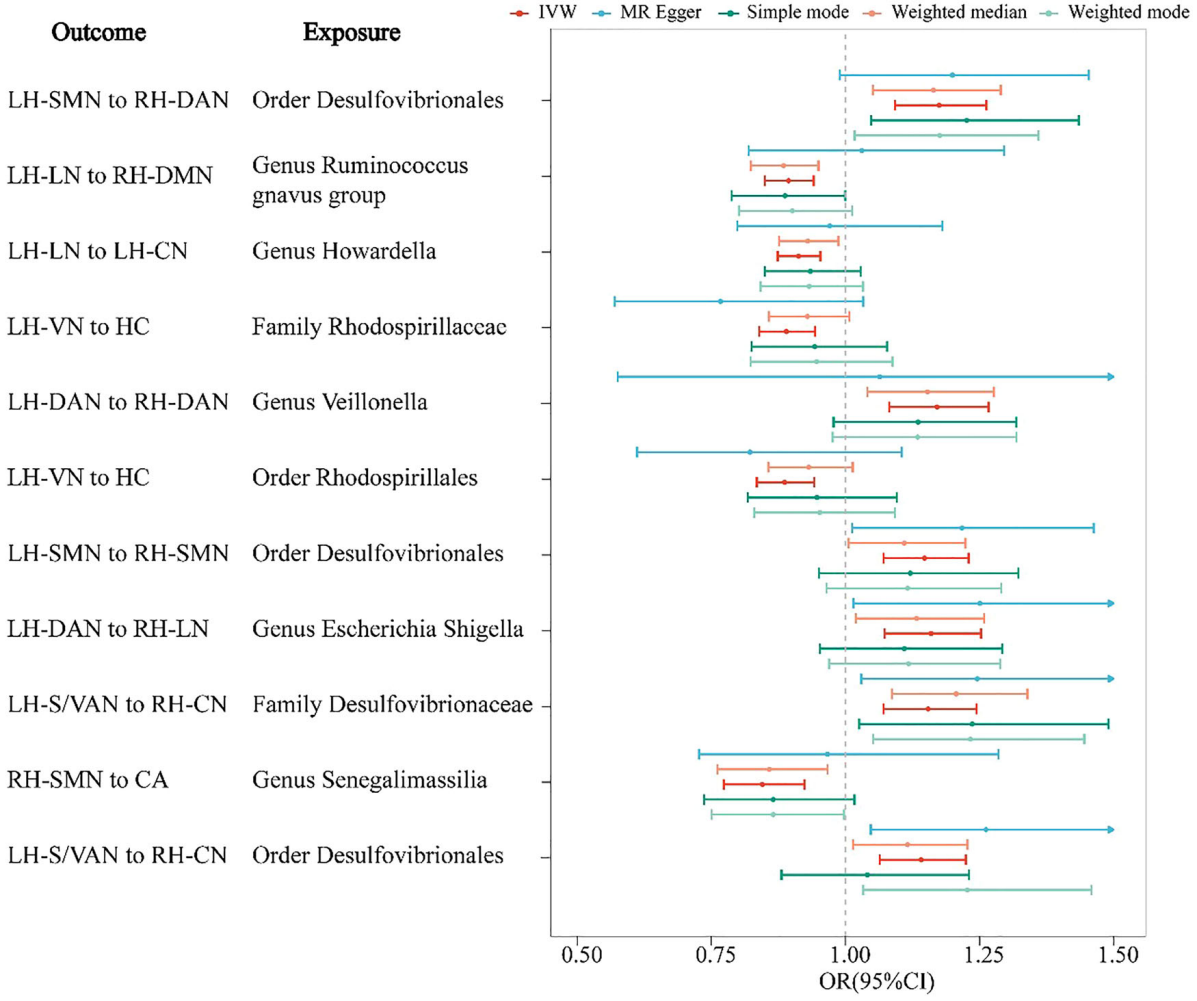
The forest plot depicts the significant association between gut microbiota and brain structural connectivity through different approaches. ***Note***: CI: Confidence Interval; OR: Odds Ratio; LH‐SMN to RH‐DAN: Left‐hemisphere somatomotor network to right‐hemisphere dorsal attention network white‐matter structural connectivity; LH‐LN to RH‐DMN: Left‐hemisphere limbic network to right‐hemisphere default mode network white‐matter structural connectivity; LH‐LN to LH‐CN: Left‐hemisphere limbic network to left‐hemisphere control network white‐matter structural connectivity; LH‐VN to HC: Left‐hemisphere visual network to hippocampus white‐matter structural connectivity; LH‐DAN to RH‐DAN: Left‐hemisphere dorsal attention network to right‐hemisphere dorsal attention network white‐matter structural connectivity; LH‐SMN to RH‐SMN: Left‐hemisphere somatomotor network to right‐hemisphere somatomotor network white‐matter structural connectivity; LH‐DAN to RH‐LN: Left‐hemisphere dorsal attention network to right‐hemisphere limbic network white‐matter structural connectivity; LH‐S/VANN to RH‐CN: Left‐hemisphere salience/ventral attention network to right‐hemisphere control network white‐matter structural connectivity; RH‐SMN to CA: Right‐hemisphere somatomotor network to caudate white‐matter structural connectivity.

A total of 11 gut microbiota were found to be significantly associated with brain structural connectivity, as determined by the Bonferroni correction with the IVW method as the primary analytical approach. Concisely, four taxa comprising order *Desulfovibrionales*, family *Desulfovibrionaceae*, genus *Escherichia Shigella*, and genus *Veillonella* were positively associated with brain structural connectivity (Table 1 and Figure 2). Notably, the order *Desulfovibrionales* demonstrated a strong positive effect on the connectivity between the left hemisphere somatomotor network and the right hemisphere dorsal attention network (OR: 1.17, 95% CI: 1.09‐1.26, *p‐value* = 1.33×10^−5^), This result was corroborated by additional MR methods (Table 1 and Figure 2). In contrast, MR analysis indicated that five gut microbiota taxa comprising genus *Senegalimassilia*, order *Rhodospirillales*, family *Rhodospirillaceae*, genus *Ruminococcus gnavus*, and genus *Howardella* were associated with decreased risk of development of brain structural connectivity (Table 1 and Figure 2). Among these, the genus *Ruminococcus gnavus* was the most notably associated with reduced connectivity between the left hemisphere limbic network and the right hemisphere default mode network (OR: 0.89, 95% CI: 0.85‐0.94, *p‐value* = 1.69×10^−5^) (Table 1 and Figure 2). Comprehensive details on the associations between gut microbiotas and brain structural connectivity are presented in Table S4.

**TABLE 1:** Significant associations of gut microbiota with the brain's structural connectivity: Findings from Mendelian randomization analyses.

Exposure	Outcome	nSNP	Methods	OR (95% CI)	*p*‐value
Order *Desulfovibrionales*	Left‐hemisphere somatomotor network to right‐hemisphere dorsal attention network white‐matter structural connectivity	12	IVW	1.175 (1.093 to 1.263)	1.33 × 10^−5^
		12	Weighted median	1.164 (1.051 to 1.290)	0.0036
		12	MR Egger	1.199 (0.989 to 1.454)	0.094
		12	Simple mode	1.226 (1.047 to 1.435)	0.028
		12	Weighted mode	1.176 (1.017 to 1.360)	0.051
	Left‐hemisphere somatomotor network to right‐hemisphere somatomotor network white‐matter structural connectivity	12	IVW	1.147 (1.071 to 1.230)	9.78 × 10^−5^
		12	Weighted median	1.110 (1.006 to 1.224)	0.038
		12	MR Egger	1.217 (1.013 to 1.463)	0.063
		12	Simple mode	1.121 (0.950 to 1.322)	0.23
		12	Weighted mode	1.116 (0.965 to 1.291)	0.17
	Left‐hemisphere salience/ventral attention network to right‐hemisphere control network white‐matter structural connectivity	12	IVW	1.141 (1.064 to 1.224)	0.00023
		12	Weighted median	1.116 (1.014 to 1.228)	0.025
		12	MR Egger	1.262 (1.047 to 1.521)	0.035
		12	Simple mode	1.041 (0.881 to 1.230)	0.65
		12	Weighted mode	1.228 (1.033 to 1.459)	0.04
Order *Rhodospirillales*	Left‐hemisphere visual network to hippocampus white‐matter structural connectivity	13	IVW	0.887 (0.834 to 0.942)	9.77 × 10^−5^
		13	Weighted median	0.931 (0.856 to 1.013)	0.099
		13	MR Egger	0.822 (0.611 to 1.105)	0.22
		13	Simple mode	0.947 (0.818 to 1.096)	0.48
		13	Weighted mode	0.952 (0.830 to 1.092)	0.5
Family *Desulfovibrionaceae*	Left‐hemisphere salience/ventral attention network to right‐hemisphere control network white‐matter structural connectivity	10	IVW	1.154 (1.070 to 1.244)	0.00019
		10	Weighted median	1.206 (1.087 to 1.339)	0.00044
		10	MR Egger	1.246 (1.029 to 1.508)	0.054
		10	Simple mode	1.236 (1.026 to 1.490)	0.053
		10	Weighted mode	1.233 (1.052 to 1.446)	0.03
Family *Rhodospirillaceae*	Left‐hemisphere visual network to hippocampus white‐matter structural connectivity	14	IVW	0.890 (0.839 to 0.943)	8.55 × 10^−5^
		14	Weighted median	0.929 (0.857 to 1.007)	0.073
		14	MR Egger	0.767 (0.569 to 1.033)	0.11
		14	Simple mode	0.943 (0.825 to 1.077)	0.4
		14	Weighted mode	0.946 (0.823 to 1.088)	0.45
Genus *Escherichia Shigella*	Left‐hemisphere dorsal attention network to right‐hemisphere limbic network white‐matter structural connectivity	9	IVW	1.160 (1.073 to 1.253)	0.00018
		9	Weighted median	1.133 (1.019 to 1.259)	0.021
		9	MR Egger	1.251 (1.015 to 1.541)	0.074
		9	Simple mode	1.110 (0.953 to 1.293)	0.22
		9	Weighted mode	1.118 (0.969 to 1.288)	0.16
Genus *Howardella*	Left‐hemisphere limbic network to left‐hemisphere control network white‐matter structural connectivity	10	IVW	0.913 (0.874 to 0.953)	4.02 × 10^−5^
		10	Weighted median	0.930 (0.876 to 0.987)	0.017
		10	MR Egger	0.971 (0.798 to 1.180)	0.77
		10	Simple mode	0.935 (0.849 to 1.029)	0.2
		10	Weighted mode	0.932 (0.841 to 1.033)	0.21
Genus *Ruminococcus gnavus group*	Left‐hemisphere limbic network to right‐hemisphere default mode network white‐matter structural connectivity	12	IVW	0.894 (0.849 to 0.941)	1.69 × 10^−5^
		12	Weighted median	0.884 (0.824 to 0.950)	0.00073
		12	MR Egger	1.030 (0.819 to 1.296)	0.8
		12	Simple mode	0.887 (0.788 to 0.999)	0.075
		12	Weighted mode	0.901 (0.801 to 1.013)	0.11
Genus *Senegalimassilia*	Right‐hemisphere somatomotor network to caudate white‐matter structural connectivity	4	IVW	0.845 (0.773 to 0.923)	0.0002
		4	Weighted median	0.858 (0.761 to 0.966)	0.012
		4	MR Egger	0.966 (0.727 to 1.285)	0.84
		4	Simple mode	0.865 (0.736 to 1.017)	0.18
		4	Weighted mode	0.865 (0.751 to 0.997)	0.14
Genus *Veillonella*	Left‐hemisphere dorsal attention network to right‐hemisphere dorsal attention network white‐matter structural connectivity	7	IVW	1.171 (1.082 to 1.267)	9.12 × 10^−5^
		7	Weighted median	1.153 (1.041 to 1.277)	0.0063
		7	MR Egger	1.064 (0.575 to 1.970)	0.85
		7	Simple mode	1.135 (0.978 to 1.318)	0.15
		7	Weighted mode	1.135 (0.976 to 1.319)	0.15

*Note*: Bacterial taxa at five levels (phylum, class, order, family, and genus).

**Abbreviations**: CI, Confidence Interval; IVW, Inverse‐variance weighted; nSNP, Number of single‐nucleotide polymorphisms; OR, Odds Ratio;

### 3.2 Sensitivity Analysis

Horizontal pleiotropy between the 11 pairs of gut microbiota and brain structural connectivity was examined by the MR Egger intercept and the global test of MR‐PRESSO. No pleiotropy was detected for any of the gut microbiota pairs (Table S5). Furthermore, heterogeneity was assessed using Cochran's Q test, and results indicated that none of the pairs exhibited heterogeneity (Table S5). Scatterplots and funnel plots for each pair of associations are given in Figure S1‐S2, while leave‐one‐out sensitivity analysis for each pair of associations is shown in Figure S3. Additionally, MR‐PRESSO analysis did not identify any significant outliers (global test *p* > 0.05, Tables S5).

### 3.3 The Causal Effect of Brain Structural Connectivity on Gut Microbiota

Following the same analytical methods, we assessed the causal relationship between brain structural connectivity and gut microbiota. Results revealed that nine brain structure connectivity phenotypes were positively associated with gut microbiota (Table S6). Among these, the left hemisphere control network to thalamus white matter structural connectivity showed an increased abundance with three gut microbiotas, including phylum *Firmicutes* (OR: 1.22, 95% CI: 1.10‐1.35, *p‐value* = 1.06×10^−4^), class *Clostridia* (OR: 1.21, 95% CI: 1.09‐1.34, *p‐value* = 2.04×10^−4^), and order *Clostridiales* (OR: 1.21, 95% CI: 1.09‐1.34, *p‐value* = 2.12×10^−4^) (Table S6). In addition, the right hemisphere default mode network to pallidum white matter structural connectivity was significantly associated with the genus *Methanobrevibacter* (OR: 1.94, 95% CI: 1.36‐2.76, *p‐value* = 2.39×10^−4^) (Table S6). Detailed results are provided in Table S6. Sensitivity analysis confirmed no heterogeneity via Cochran's Q test, and no evidence of horizontal pleiotropy was detected by MR‐Egger's intercept test and MR‐PRESSO global test (Table S7).

### 3.4 Mediatory Role of Cytokine in Gut‐Brain Connectivity

#### 3.4.1 Effect of Gut Microbiota on Inflammatory Cytokines

Using Mendelian randomization with Bonferroni correction, our study found 26 significant associations between gut microbiota and inflammatory cytokines across various taxonomic levels, that is, one phylum, one class, three orders, six families, and 15 genera. Among them, the phylum *Euryarchaeota* was a risk factor for IL‐2 (OR: 1.19, 95% CI: 1.09‐1.31, *p‐value* = 1.34×10^−4^). Additionally, class *Verrucomicrobiae* was a risk factor for PDGF‐BB (OR: 1.23, 95% CI: 1.11‐1.37, *p‐value* = 1.09×10^−4^). Furthermore, among the three orders, two were identified as risk factors, that is, Verrucomicrobiales for PDGF‐BB (OR: 1.23, 95% CI: 1.11‐1.37, *p‐value* = 1.09×10^−4^) and Enterobacteriales for IL‐1RA (OR: 1.55, 95% CI: 1.23‐1.94, *p‐value* = 1.84×10^−4^), whereas Lactobacillales exhibited a protective effect for B‐NGF (OR 0.71, 95% CI: 0.60‐0.85, *p‐value* = 1.18×10^−4^). In addition, four of the six family‐level taxa were identified as risk factors, that is, *Porphyromonadaceae* for VEGF (OR: 1.35, 95% CI: 1.16‐1.58, *p‐value* = 1.08×10^−4^), *Verrucomicrobiaceae* for PDGF‐BB (OR: 1.23, 95% CI: 1.11‐1.37, *p‐value* = 1.10×10^−4^), *Family XIII* for M‐CSF (OR: 1.70, 95% CI: 1.29‐2.23, *p‐value* = 1.37×10^−4^), *Enterobacteriaceae* for IL‐1RA (OR: 1.55, 95% CI: 1.23‐1.94, *p‐value* = 1.84×10^−4^). And two families showed protective effects, that is, *Defluviitaleaceae* for PDGF‐BB (OR: 0.82, 95% CI: 0.75‐0.91, *p‐value* = 1.15×10^−4^) and *Oxalobacteraceae* for MIG (OR: 0.84, 95% CI: 0.77‐0.92, *p‐value* = 1.30×10^−4^). Finally, among the 15 genera, *Enterorhabdus* was identified as the most significant risk factor for MCP‐3 (OR: 1.92, 95% CI: 1.38‐2.67, *p‐value* = 1.05×10^−4^). Additional risk factors included *Butyricicoccus* for TNF‐B (OR: 2.23, 95% CI: 1.47‐3.39, *p‐value* = 1.66×10^−4^), *Paraprevotella* for TNF‐B (OR: 1.38, 95% CI: 1.17‐1.63, *p‐value* = 1.33×10^−4^), *Streptococcus* for IL‐7 (OR: 1.36, 95% CI: 1.16‐1.59, *p‐value* = 1.64×10^−4^), *Ruminococcaceae UCG002* for MIG (OR: 1.27, 95% CI: 1.13‐1.44, *p‐value* = 1.26×10^−4^), and *Akkermansia* for PDGF‐BB (OR: 1.23, 95% CI: 1.11‐1.37, *p‐value* = 1.09×10^−4^). Moreover, Protective factors included *Parasutterella* for IL‐4 (OR: 0.85, 95% CI: 0.78‐0.92, *p‐value* = 1.44×10^−4^), *Haemophilus* for MCP‐1/MCAF (OR: 0.84, 95% CI: 0.77‐0.92, *p‐value* = 1.70×10^−4^), *Oscillibacter* for TRAIL (OR: 0.83, 95% CI: 0.75‐0.92, *p‐value* = 1.96×10^−4^), *Oxalobacter* for IL‐18 (OR: 0.83, 95% CI: 0.75‐0.91, *p‐value* = 1.32×10^−4^), *Ruminiclostridium5* for IL‐12_P70 (OR: 0.78, 95% CI: 0.69‐0.89, *p‐value* = 1.83×10^−4^), *Ruminococcaceae UCG013* for MIP‐1B (OR: 0.78, 95% CI: 0.68‐0.89, *p‐value* = 1.95×10^−4^), *Ruminiclostridium9* for MCP‐1/MCAF (OR: 0.75, 95% CI: 0.65‐0.87, *p‐value* = 1.07×10^−4^), *Ruminiclostridium6* for B‐NGF (OR: 0.74, 95% CI: 0.64‐0.86, *p‐value* = 1.34×10^−4^), and *Eubacterium xylanophilum* for RANTES (OR: 0.70, 95% CI: 0.59‐0.85, *p‐value* = 1.66×10^−4^). All results are given in Table S8.

#### 3.4.2 Effect of Inflammatory Cytokines on Brain Structural Connectivity

After determined by the Bonferroni correction, a total of 14 significant causal associations between inflammatory cytokines and brain structural connectivity traits were identified. Among these, RANTES was identified as a risk factor for seven brain structural connectivity. The most significant association was found between left hemisphere salience/ventral attention network and right hemisphere visual network white matter structural connectivity (OR: 1.11, 95% CI: 1.07‐1.16, *p‐value* = 2. 58×10^−7^). Additional notable associations include the left hemisphere dorsal attention network to left hemisphere limbic network white matter structural connectivity (OR: 1.08, 95% CI: 1.04‐1.13, *p‐value* = 2. 14×10^−4^), left hemisphere limbic network to hippocampal white matter structural connectivity (OR: 1.09, 95% CI: 1.04‐1.14, *p‐value* = 2. 66×10^−4^), left hemisphere limbic network to caudate nucleus white matter structural connectivity (OR: 1.07, 95% CI: 1.03‐1.12, *p‐value* = 5. 13×10^−4^), right hemisphere somatomotor network to right hemisphere salience/introspective attention network white matter structural connectivity (OR: 1.07, 95% CI: 1.03‐1.11, *p‐value* = 8. 31×10^−4^), left hemisphere visual network to right hemisphere somatomotor network white matter structural connectivity (OR: 1.08, 95% CI: 1.03‐1.14, *p‐value* = 1. 07×10^−3^), and left hemisphere default mode network with right hemisphere somatomotor network white matter structural connectivity (OR: 1.07, 95% CI: 1.03‐1.11, *p‐value* = 1.14×10^−3^). Additionally, HGF was associated as a protective factor for five brain structure connectivity traits, notably between the right hemisphere visual network and hippocampal white matter structure connectivity (OR: 0. 90, 95% CI: 0.85‐0.95, *p‐value* = 4. 23×10^−4^), left hemisphere visual network and hippocampal white matter structure connectivity (OR: 1.07, 95% CI: 1.03‐1.11, *p‐value* = 1. 14×10^−3^), left hemisphere visual network and hippocampus white matter structure connectivity (OR: 0.90, 95% CI: 0.86‐0.96, *p‐value* = 4.66×10^−4^), left hemisphere limbic network to left hemisphere limbic network white matter structure connectivity (OR: 0.91, 95% CI: 0.86‐0.96, *p‐value* = 7.88×10^−4^), and right hemisphere dorsal attention network to right hemisphere limbic network white matter structure connectivity (OR: 0.91, 95% CI: 0.86‐0.96, *p‐value* = 1. 09×10^−3^). Furthermore, PDGF‐BB served as a protective factor for white matter structural connectivity from the left hemisphere limbic network to the right hemisphere dorsal attention network (OR: 0.94, 95% CI: 0.91‐0.97, *p‐value* = 3. 18×10^−4^). Conversely, IL‐13 was identified as a risk factor for the left hemisphere default mode network to caudate white matter structural connectivity (OR: 1.05, 95% CI: 1.02‐1.07, *p‐value* = 3. 60×10^−4^), and IL‐12_P70 was identified as a risk factor for connectivity of the right hemisphere salience/ventral attention network with white matter structures in the amygdala (OR: 1.06, 95% CI: 1.02‐1.09, *p‐value* = 1.11×10^−3^). All MR results are summarized in Table S9.

#### 3.4.3 Mediation Analysis of Gut Microbiota on Brain Structural Connectivity

By performing an MR mediation analysis, we examined the role of inflammatory cytokines as mediators between gut microbiota and brain structural connectivity and identified 10 inflammatory cytokines that mediate the causal relationship between gut microbiota and brain structural connectivity (Table 2). Concisely, the results revealed that inflammatory cytokines, including RANTES, HGF, and IL‐13, play a significant mediatory role in the relationship between gut microbiota and white matter structural connectivity in different brain regions (Table 2). For instance, the genus *Blautia* significantly influenced the connectivity from the right hemisphere somatomotor to the limbic network with HGF as the mediator, contributing a 9.54% mediation proportion (Table 2). Similarly, the Lachnospiraceae NK4A136 group was linked to the connectivity between the left hemisphere salience/ventral attention network and the right hemisphere visual network, mediated by RANTES with a 30.18% effect (Table 2).

**TABLE 2:** The mediation effect of gut microbiota on brain structural connectivity via inflammatory cytokines.

Exposure	Mediator	Outcome	Total effect	Direct effect A	Direct effect B	Mediation effect	Mediated proportion (%)
			β (95% CI)	β (95% CI)	β (95% CI)	β (95% CI)	
Family *Lactobacillaceae*	G‐CSF	Left‐hemisphere control network to accumbens white‐matter structural connectivity	0.065	0.134	0.062	0.008	12.78
			(0.007, 0.122)	(0.048, 0.221)	(0.018, 0.105)	(0.000, 0.016)	
Genus *Lachnospiraceae UCG010*			0.102	0.21	0.062	0.013	12.63
			(0.029, 0.175)	(0.073, ‐0.346)	(0.018, 0.105)	(0.000, 0.025)	
Genus *Desulfovibrio*			−0.122	−0.264	0.062	−0.016	13.36
			(‐0.229, ‐0.015)	(‐0.393, ‐0.135)	(0.018, 0.105)	(‐0.030, ‐0.002)	
Family *Victivallaceae*	HGF	Left‐hemisphere limbic network to left‐hemisphere default mode network white‐matter structural connectivity	−0.042	0.096	−0.105	−0.01	23.83
			(‐0.080, ‐0.004)	(0.037, 0.154)	(‐0179, ‐0.032)	(‐0.019, ‐0.001)	
Genus *Blautia*		Right‐hemisphere somatomotor network to right‐hemisphere limbic network white‐matter structural connectivity	−0.397	0.538	−0.07	−0.038	9.54
			(‐0.763, ‐0.031)	(0.219, 0.857)	(‐0.127, ‐0.014)	(‐0.076, ‐0.000)	
Family *Clostridiales vadin BB60 group*	IL‐13	Right‐hemisphere somatomotor network to right‐hemisphere default mode network white‐matter structural connectivity	−0.076	−0.221	0.033	−0.007	9.58
			(‐0.139, ‐0.013)	(‐0.346, ‐0.096)	(0.009, 0.057)	(‐0.014, ‐0.001)	
Genus *Paraprevotella*		Left‐hemisphere somatomotor network to left‐hemisphere default mode network white‐matter structural connectivity	0.06	0.184	0.033	0.006	10.09
			(0.012, 0.107)	(0.075, 0.293)	(0.009, 0.056)	(0.000, 0.012)	
Genus *Phascolarctobacterium*		Left‐hemisphere salience/ventral attention network to caudate white‐matter structural connectivity	0.098	0.264	0.036	0.01	9.74
			(0.020, 0.177)	(0.098, 0.431)	(0.009, 0.064)	(0.000, 0.019)	
Genus *Oxalobacter*	IL‐18	Left‐hemisphere visual network to thalamus white‐matter structural connectivity	−0.047	−0.187	0.037	−0.007	14.51
			(‐0.093, ‐0.001)	(‐0.282, ‐0.091)	(0.010, 0.063)	(‐0.013, ‐0.001)	
Genus *Ruminiclostridium6*	IP‐10	Right‐hemisphere somatomotor network to amygdala white‐matter structural connectivity	−0.066	−0.231	0.055	−0.013	19.17
			(‐0.130, ‐0.002)	(‐0.381, ‐0.082)	(0.015, 0.095)	(‐0.025, 0.000)	
Genus *Sellimonas*		Right‐hemisphere default mode network to accumbens white‐matter structural connectivity	0.07	0.184	0.051	0.009	13.41
			(0.026, 0.114)	(0.082, 0.286)	(0.014, 0.088)	(0.001, 0.018)	
Genus *Ruminococcaceae UCG005*	PDGF‐BB	Left‐hemisphere limbic network to right‐hemisphere dorsal attention network white‐matter structural connectivity	0.070	−0.165	−0.063	0.010	14.73
			(0.002, 0.139)	(‐0.264, ‐0.066)	(‐0.097, ‐0.029)	(0.002, 0.019)	
Genus *Sellimonas*	MIG	Right‐hemisphere somatomotor network to accumbens white‐matter structural connectivity	0.048	0.147	0.051	0.008	15.55
			(0.002, 0.094)	(0.056, 0.238)	(0.014, 0.089)	(0.000, 0.015)	
Genus *Ruminococcaceae UCG002*		Left‐hemisphere visual network to putamen white‐matter structural connectivity	0.065	0.243	0.044	0.011	16.40
			(0.006, 0.124)	(0.119, 0.367)	(0.013, 0.075)	(0.001, 0.020)	
Family *Defluviitaleaceae*	RANTES	Right‐hemisphere salience/ventral attention network to right‐hemisphere control network white‐matter structural connectivity	−0.072	−0.265	0.051	−0.013	18.65
			(‐0.129, ‐0.015)	(‐0.416, ‐0.113)	(0.012, 0.089)	(‐0.026, ‐0.001)	
Family *Rhodospirillaceae*		Left‐hemisphere visual network to right‐hemisphere dorsal attention network white‐matter structural connectivity	−0.074	−0.21	0.058	−0.012	16.68
			(‐0.134, ‐0.013)	(‐0.352, ‐0.067)	(0.018, 0.099)	(‐0.024, ‐ 0.000)	
Genus *Lachnospiraceae NK4A136 group*		Left‐hemisphere default mode network to right‐hemisphere visual network white‐matter structural connectivity	−0.084	−0.191	0.065	−0.012	14.67
			(‐0.147, ‐0.020)	(‐0.338, ‐0.044)	(0.023, 0.106)	(‐0.025, 0.000)	
Genus *Eubacterium xylanophilum group*		Left‐hemisphere somatomotor network to left‐hemisphere default mode network white‐matter structural connectivity	−0.112	−0.351	0.05	−0.017	15.6
			(‐0.222, ‐0.000)	(‐0.533, ‐0.168)	(0.011, 0.088)	(‐0.034, ‐0.001)	
Genus *Lachnospiraceae NK4A136 group*		Left‐hemisphere salience/ventral attention network to right‐hemisphere visual network white‐matter structural connectivity	−0.068	−0.191	0.108	−0.021	30.18
			(‐0.135, ‐0.001)	(‐0.338, ‐0.044)	(0.067, 0.149)	(‐0.0383, ‐0.003)	
Genus *Lachnospiraceae NK4A136 group*		Left‐hemisphere somatomotor network to right‐hemisphere default mode network white‐matter structural connectivity	−0.086	−0.191	0.062	−0.012	13.79
			(‐0.150, ‐0.022)	(‐0.338, ‐0.044)	(0.023, 0.101)	(‐0.024, ‐ 0.000)	
Genus *Lachnospiraceae NK4A136 group*		Left‐hemisphere salience/ventral attention network to right‐hemisphere control network white‐matter structural connectivity	−0.062	−0.191	0.06	−0.011	18.46
			(‐0.124, ‐0.001)	(‐0.338, ‐0.044)	(0.023, 0.098)	(‐0.023, 0.000)	
Genus *Ruminococcaceae UCG003*	TNF‐B	Right‐hemisphere somatomotor network to caudate white‐matter structural connectivity	−0.116	−0.461	0.04	−0.018	15.66
			(‐0.190, ‐0.043)	(‐0.800, ‐0.122)	(0.015, 0.064)	(‐0.036, ‐0.001)	
Family *Victivallaceae*	TRAIL	Right‐hemisphere limbic network to amygdala white‐matter structural connectivity	0.052	0.095	0.04	0.004	7.34
			(0.004, 0.101)	(0.036, 0.154)	(0.010, 0.071)	(0.000, 0.008)	

*Note*: ‘Total effect’ indicates the effect of gut microbiota on brain structural connectivity, ‘Direct effect A’ indicates the effect of gut microbiota on inflammatory cytokines, ‘Direct effect B' indicates the effect of inflammatory cytokines on brain structural connectivity, and ‘Mediation effect’ indicates the effect of gut microbiota on brain structural connectivity via inflammatory cytokines. Total effect, direct effect A, and direct effect B were derived by IVW; mediation effect was derived by using the delta method. All statistical tests were two‐sided; *P* < 0.05 was considered significant.

The mediation effects varied across different brain regions, with RANTES frequently acting as the mediator, particularly in the interactions involving the left‐hemisphere somatomotor network. Notably, cytokines such as TNF‐β, G‐CSF, and MIG were implicated in mediating the relationship between specific microbial taxa and brain structural connectivity, such as genus *Ruminococcaceae UCG003* and genus *Lachnospiraceae UCG010*, and white‐matter connectivity in brain regions like the accumbens and amygdala (Table 2). The mediation effects were generally moderate, with the mediated proportion of total effects ranging from 10% to 30% (Table 2). These findings highlight the crucial role of inflammatory cytokines, such as RANTES, HGF, and IL‐13, as intermediaries in the gut‐brain axis, influencing the structural connectivity between different brain regions. Detailed results are given in Table S10.

### 3.5 GO, KEGG, and Reactome Analysis of Inflammatory Cytokines

**FIGURE 3:**
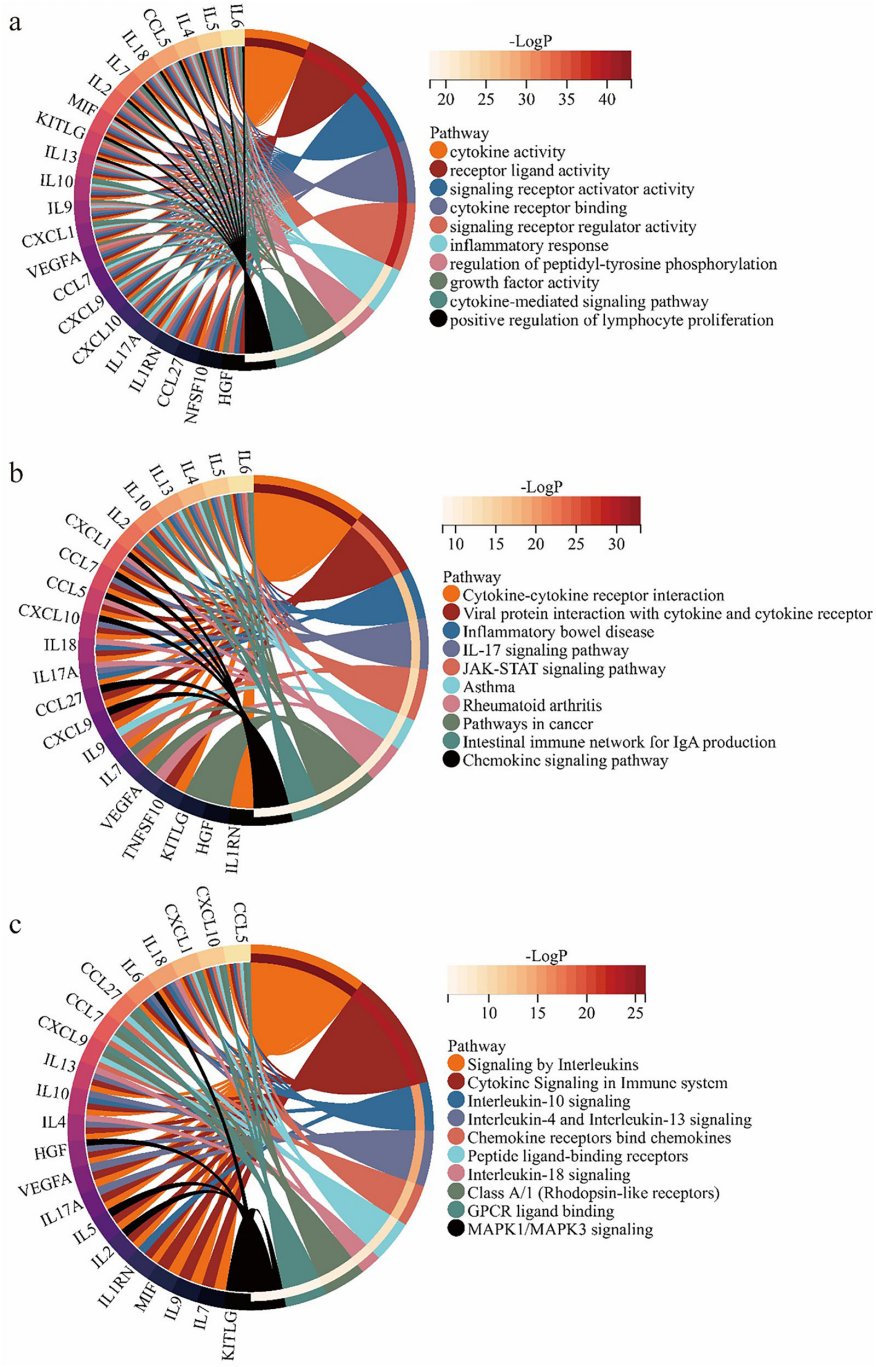
Enrichment analysis for inflammatory cytokines. (a) GO analysis, (b) KEGG analysis, (c) Reactome analysis.

GO, KEGG, and Reactome pathway enrichment analyses were performed to elucidate the potential mechanisms linking gut microbiota‐mediated inflammatory cytokines' effect on brain structure connectivity. GO analysis revealed significant enrichment in pathways related to cytokine activity, receptor‐ligand interactions, and cytokine‐mediated signaling, emphasizing the critical role of cytokine‐receptor interactions in mediating downstream signaling processes (Figure 3a). KEGG pathway analysis identified key signaling cascades, including the JAK‐STAT and IL‐17 pathways, highlighting their involvement in inflammatory diseases like inflammatory bowel disease (IBD) (Figure 3b). The reactome analysis further pinpointed pathways, including interleukin signaling, chemokine receptor binding, and MAPK signaling, suggesting that these inflammatory factors influence neuronal activity and connectivity through these pathways (Figure 3c). Based on our MR mediation analysis, we identified RANTES (CCL5), HGF, and IL‐13 as the most robust cytokine mediators underlying the gut microbiota–brain structural connectivity axis. These cytokines consistently exhibited significant mediation across multiple brain networks, with the proportion mediated ranging from 9.54% to 30.18%. Pathway enrichment analyses further revealed that these cytokines are core components of highly relevant KEGG pathways, including the cytokine–cytokine receptor interaction, chemokine signaling, JAK–STAT, and IL‐17 pathways. These findings underscore the pivotal role of inflammatory signaling networks in mediating the gut‐brain axis and offer valuable insights into the molecular mechanisms underlying brain structural connectivity changes driven by gut microbiota dysbiosis. The complete results of GO, KEGG, and analysis are presented in Table S11–S13, respectively.

## 4 Discussion

Given the significant impact of the microbiota‐gut‐brain axis, it is crucial to systematically investigate the causal relationship between gut microbiota and brain structural connectivity. To the best of our knowledge, this is the first study to comprehensively evaluate the causal associations between gut microbiota, inflammatory cytokines, and brain structural connectivity. In this study, we employed bidirectional two‐sample MR analysis to rigorously examine the causal links between gut microbiota and brain structural alterations. Additionally, our findings provide a systematic investigation of the mediating effects of inflammatory cytokines in the relationship between gut microbiota and brain structural connectivity. After applying Bonferroni correction, we identified 11 gut microbiota taxa with significant causal relationships to brain structural connectivity, highlighting their potential roles within the microbiota‐gut‐brain axis. This research substantially enhances our understanding of the microbiota‐gut‐brain axis.

In previous studies, Hu et al. (2024) and Huang et al. (2023) performed MR analysis to investigate the effects of gut flora on subcortical brain structures and the rate of change of brain structures, respectively. Our study differs from theirs in two significant aspects. Firstly, while the studies by Hu et al. (2024) and Huang et al. (2023) focused on specific brain structures, our study utilizes MRI‐based GWAS data encompassing neural connectivity across the entire brain. MRI‐based neural connectivity data provides a comprehensive view of connectivity within the whole brain network, offering a more comprehensive understanding of brain activity compared to focusing solely on subcortical structures or their rate of change. These data not only capture structural connectivity but also reflect functional connectivity, enabling a more thorough analysis of the relationship between functional and structural connectivity patterns and the interactions between gut flora and various brain regions. Secondly, our MR analysis was more comprehensive, incorporating both two‐sample MR and additional sensitivity analyses, such as horizontal pleiotropy assessment and reverse MR analysis. Furthermore, we performed a mediation analysis to investigate the role of inflammatory cytokines, allowing us to systematically explore the interactions between gut flora and neural connectivity of the brain. In summary, our study offers a more detailed and thorough exploration of the relationship between gut flora and neural connectivity, employing advanced methodologies to understand the complex interactions within the brain's entire connectivity network.

Our findings reveal patterns and mechanisms of gut flora‐induced brain alterations analyzed through a large population of GWAS data. The family *Desulfovibrionaceae* and order *Desulfovibrionales*, which are cognate with the genus *Desulfovibrio*, can lead to alterations in the white‐matter structural connectivity responsible for connections with attention and somatomotor (Table 1 and Figure 2). Additionally, our study underscored the importance of mediators such as RANTES and G‐CSF in modulating gut‐brain interactions, emphasizing their potential to influence brain structural connectivity, especially through the genus *Desulfovibrio* and family *Defluviitaleaceae* (Table 2). Notably, previous studies have reported increased levels of the genus *Desulfovibrio* in patients with Parkinson's Disease (Nie et al. 2023) compared to healthy controls, correlating positively with disease severity, suggesting a potential pathogenic role. Furthermore, significant correlations between the order *Desulfovibrionales* and clinical stroke scales (Li et al. 2022b) reinforce the clinical relevance of gut‐brain interactions. Research indicates that *Desulfovibrio*’s production of excess hydrogen sulfide in the gastrointestinal tract may induce brain damage and neurological symptoms, with elevated plasma sulfide levels being associated with brain atrophy and reduced white matter integrity, potentially leading to neurologically related diseases (Haouzi et al. 2020; Panthi et al. 2018). Furthermore, G‐CSF, a cytokine that plays a key role in the production and activation of granulocytes, has been shown to offer long‐term neuroprotection by preventing brain atrophy and inducing somatic development, as demonstrated in numerous ischemic rodent models (Modi et al. 2020). G‐CSF also has potential therapeutic applications in neurodegenerative disorders such as Parkinson's disease (PD), intracerebral hemorrhage, experimental allergic encephalomyelitis, cerebral ischemia, spinal cord injury, and Alzheimer's disease (AD) (Rahi et al. 2021). The gut microbiota can activate immune cells through the gut‐immune axis, further linking immune modulation with changes in brain structural connectivity (Forrest et al. 1994). Therefore, we hypothesize that excessive *Desulfovibrio* in the gut contributes to the development of neurological disease through the production of hydrogen sulfide, which reduces the systemic inflammatory response and may inhibit G‐CSF production in immune cells. This, in turn, could lead to neurodegenerative disorders, brain atrophy, and reduced white matter integrity (Reekes et al. 2023).

Our study also reveals that *Ruminococcus gnavus* acts as a protective factor for white matter connectivity between the limbic network and the default mode network (Figure 2). Abnormal connectivity between these networks can lead to impaired attention, control, and emotional regulation, potentially contributing to conditions such as attention deficit hyperactivity disorder (ADHD) (Wan et al. 2020) and mood disorders (Tomasi and Volkow 2012). *Ruminococcus gnavus*, a member of the phylum *Firmicutes*, is a common human gut symbiont (Crost et al. 2023). Furthermore, studies suggest that *Ruminococcus gnavus* alters the gut environment by reducing sialic acid derivatives, which may influence brain function via the gut‐brain axis (Marizzoni et al. 2023). This bacterium might also enhance synaptic plasticity, thereby improving cognitive functions such as learning and memory. Additionally*, Ruminococcus gnavus* secretes SCFAs (Crost et al. 2023), which have neuroprotective effects on neurons (Hamamah et al. 2022) and can reduce anxiety and depression (Feng et al. 2023). Collectively, these findings highlight the multifaceted influence of *Ruminococcus gnavus* on brain function, underscoring its potential role in neurological disorders. However, the precise relationship between *Ruminococcus gnavus* and brain structural connectivity requires further investigation.

Through reverse Mendelian randomization analysis, we uncovered the potential regulatory role of various brain structural connectivity in shaping gut microbiota, particularly the influence of thalamic, fornix, and amygdala white matter on enhancing the abundance of *Firmicutes* and its class *Clostridia*. This finding broadens our understanding of the gut‐brain interaction and highlights the critical role of neuro‐gut pathways in regulating gut microbial composition. The thalamus, fornix white matter, and amygdala white matter play key roles in neural regulation and emotion processing. Their complex neural circuits and interactions may influence gut microbiota composition by regulating the endocrine system, autonomic nervous system, and emotional regulation functions. Specifically, this neuro‐gut regulation is thought to be mediated by two primary systems. First, the autonomic nervous system, particularly the vagus nerve, serves as a direct communication highway between the brain and gut. Alterations in the structural connectivity of brain regions like the amygdala could modulate vagal nerve signals (Cryan and Dinan 2012), which in turn influence gut motility, secretion of mucus and gastric acid, and the integrity of the intestinal barrier. These changes to the gut environment exert selective pressure on microbial communities, potentially favoring the growth of specific microbial communities (Carabotti et al. 2015). Second, the neuroendocrine system, primarily through the hypothalamic‐pituitary‐adrenal axis, provides a hormonal pathway for this regulation. Dysregulation in brain circuits involved in emotion processing can alter the release of stress hormones like cortisol (Zheng et al. 2016). Cortisol can directly impact the gut by modulating local immune responses and altering the metabolic environment, thereby shaping the composition of the gut microbiota (Bercik et al. 2012). Previous research, including studies by Labus et al. (2019), has examined the interactions among gut microbial abundance, gastrointestinal sensorimotor function, and brain functional network indicators in irritable bowel syndrome. Their findings revealed that connectivity between subcortical (thalamus, caudate nucleus, and putamen) and cortical (primary and secondary somatosensory cortex) regions mediated the subnetwork links of *Clostridium* cluster XIVa (*Clostridium coccoides*) and genus *Coprococcus*. However, the direct impact of brain structural connectivity on gut microbiota remains unclear, necessitating further in‐depth research.

Our enrichment analyses highlighted the pivotal role of cytokine‐mediated pathways, such as JAK‐STAT, IL‐17 pathways, and MAPK signaling, in connecting gut microbiota dysbiosis with alterations in brain connectivity. These findings support previous research that highlights the involvement of inflammatory pathways in modulating neuroinflammatory responses and neural plasticity. For example, the JAK‐STAT pathway has been shown to play a key role in microglial activation and neurodegenerative processes (Panda et al. 2024). Moreover, the MAPK pathway is involved in regulating synaptic signaling and neuronal survival (Thomas and Huganir 2004). The enrichment of interleukin and chemokine‐related pathways further suggests that inflammatory factors, particularly cytokines and chemokines, act as essential mediators in the gut‐brain axis, potentially influencing neurogenesis, synaptic connectivity, and even cognitive functions.

While our study offers preliminary evidence supporting the hypothesis of brain‐gut interaction, several limitations persist. Firstly, to ensure sufficient statistical power when analyzing the highly polygenic traits of the gut microbiota, we adopted a relatively lenient significance threshold of *p* < 1 × 10^−5^ for instrument selection. Although this approach is commonly used in microbiome‐related MR studies, it is less stringent than the conventional genome‐wide significance threshold (*p* < 5 × 10^−8^) and may increase the risk of incorporating weak instruments. Future replications with larger GWAS summary statistics may allow for the use of stricter thresholds. Second, although Mendelian randomization is a powerful tool for reducing the influence of confounding variables, it cannot entirely rule out the presence of other potential confounders. Meanwhile, MR analysis helps assess causal relationships between exposure and outcome, but it cannot replace clinical trials, especially in objective domains. Therefore, further research is needed to confirm the proposed association between gut flora and neural connectivity in the brain. Third, our mediation analysis has specific limitations. A key assumption is that the association between the mediators and the outcome is not influenced by unmeasured confounders. While the MR design mitigates many such risks, we cannot rule out that complex pleiotropic pathways or an unknown factor could independently affect both a mediator and the outcome, potentially biasing the estimated mediation effect. Fourth, our study has not yet explored the molecular mechanisms underlying the neural‐gut interaction, which remains an important area for future research. Lastly, a deeper understanding of how genetic and environmental factors influence this interaction is essential for advancing our knowledge.

## 5 Conclusion

This study uncovers significant causal associations between gut microbiota and brain structural connectivity, highlighting the intricate role of inflammatory cytokines in mediating these relationships. We identified 11 causal relationships between the gut microbiota and brain structural connectivity, with 10 inflammatory cytokines mediating the relationship, suggesting a complex interaction between the two. In addition, nine reverse causal relationships were identified linking brain structural connectivity to alterations in the composition of the gut microbiota. Mediation analyses revealed that RANTES receptor levels mediated up to 30.18% of the relationship between the genus *Lachnospiraceae NK4A136* group and the left hemisphere salience/ventral attention network and the right hemisphere visual network. These findings not only deepen our understanding of the mechanisms of the gut‐brain axis but also provide new insights into the treatment of neurological disorders and promote innovative approaches to health promotion and disease management.

## Author Contributions

**Qianling Guo**: conceptualization, design, data curation, formal analysis, visualization, writing – original draft, writing – review and editing. **Dongli Yang**: conceptualization, design, data curation, formal analysis, visualization, writing – original draft, writing – review and editing. **Aamir Fahira**: validation, writing – review and editing. **Qiusheng Zhong**: writing – review and editing, supervision, funding acquisition. **Jiahao Yang**: data curation, formal analysis, writing – original draft. **Kai Zhuang**: writing – review and editing. **Ying Wen**: validation. **Zhuolun Tang**: visualization. **Zunnan Huang**: conceptualization, design, writing – review and editing, supervision, funding acquisition. All authors contributed to the article and approved the submitted version.

## Conflicts of Interest

The authors declare no conflicts of interest.

## Peer Review

The peer review history for this article is available at https://publons.com/publon/10.1002/brb3.70980.

## Supporting information




**Supplementary Materials**: brb370980‐sup‐0001‐FigureS1‐S3.docx

**Supplementary Materials**:brb370980‐sup‐0002‐TableS1‐S13.xlsx

## Data Availability

This study analyzed publicly available datasets. The GWAS summary statistics for brain structural connectivity are accessible in the GWAS Catalog (https://www.ebi.ac.uk/gwas/). Summary statistics for gut microbiota can be found at the MiBioGen consortium (https://mibiogen.gcc.rug.nl/). The GWAS summary statistical data for Inflammatory cytokines are available at https://data.bris.ac.uk/data/dataset/.
