# Supplementary material for: Dissecting Causal Relationships Between Gut Microbiota Imbalance, Inflammatory Cytokines, and Structural Connectivity in the Brain: A Mendelian Randomization Study
Source: Brain Behav. 2025 Oct 29;15(11):e70980. doi: 10.1002/brb3.70980 (PMC12571987; doi:10.1002/brb3.70980)
Supplement: Supplementary file 1 — Supplementary Materials: brb370980‐sup‐0001‐FigureS1‐S3.docx [file BRB3-15-e70980-s001.docx]

**Dissecting Causal Relationships Between gut microbiota imbalance, inflammatory cytokines, and structural connectivity in the brain: A Mendelian Randomization Study**

**Supplementary Contents**

**Figure S1.** Scatter plot of a two sample Mendelian randomization study of gut microbiota and brain structural connectivity.

**Figure S2.** Funnel plot of a two-sample Mendelian randomization study of gut microbiota and brain structural connectivity.

**Figure S3.** Forest plot of a two-sample Mendelian randomization study of gut microbiota and brain structural connectivity.


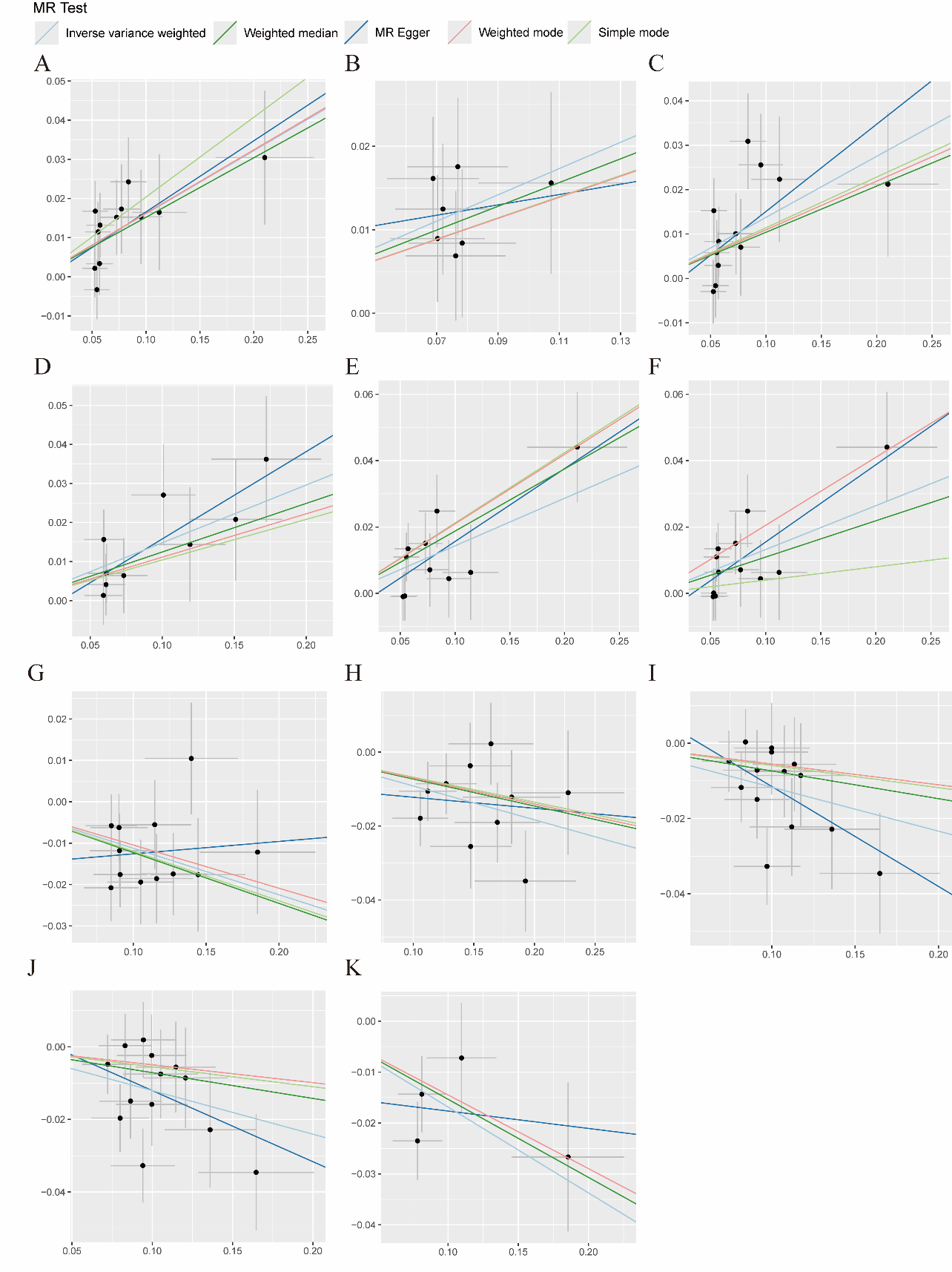


**Figure S1.** Scatter plot of a two sample Mendelian randomization study of gut microbiota and brain structural connectivity. **(A)** Order *Desulfovibrionales* on Left−hemisphere somatomotor network to right−hemisphere dorsal attention network white−matter structural connectivity. **(B)** Genus *Veillonella* on Left−hemisphere dorsal attention network to right−hemisphere dorsal attention network white−matter structural connectivity. **(C)** Order *Desulfovibrionales* on SNP effect on Left−hemisphere somatomotor network to right−hemisphere somatomotor network white−matter structural connectivity. **(D)** Genus *Escherichia Shigella* on Left−hemisphere dorsal attention network to right−hemisphere limbic network white−matter structural connectivity. **(E)** Family *Desulfovibrionaceae* on Left−hemisphere salience/ventral attention network to right−hemisphere control network white−matter structural connectivity. **(F)** Order *Desulfovibrionales* on Left−hemisphere salience/ventral attention network to right−hemisphere control network white−matter structural connectivity. **(G)** Genus *Ruminococcus gnavus* group on Left−hemisphere limbic network to right−hemisphere default mode network white−matter structural connectivity. **(H)** Genus *Howardella* on Left−hemisphere limbic network to left−hemisphere control network white−matter structural connectivity. **(I)** Family *Rhodospirillaceae* on Left−hemisphere visual network to hippocampus white−matter structural connectivity. **(J)** Order *Rhodospirillales* on Left−hemisphere visual network to hippocampus white−matter structural connectivity. **(K)** Genus *Senegalimassilia* on Right−hemisphere somatomotor network to caudate white−matter structural connectivity.


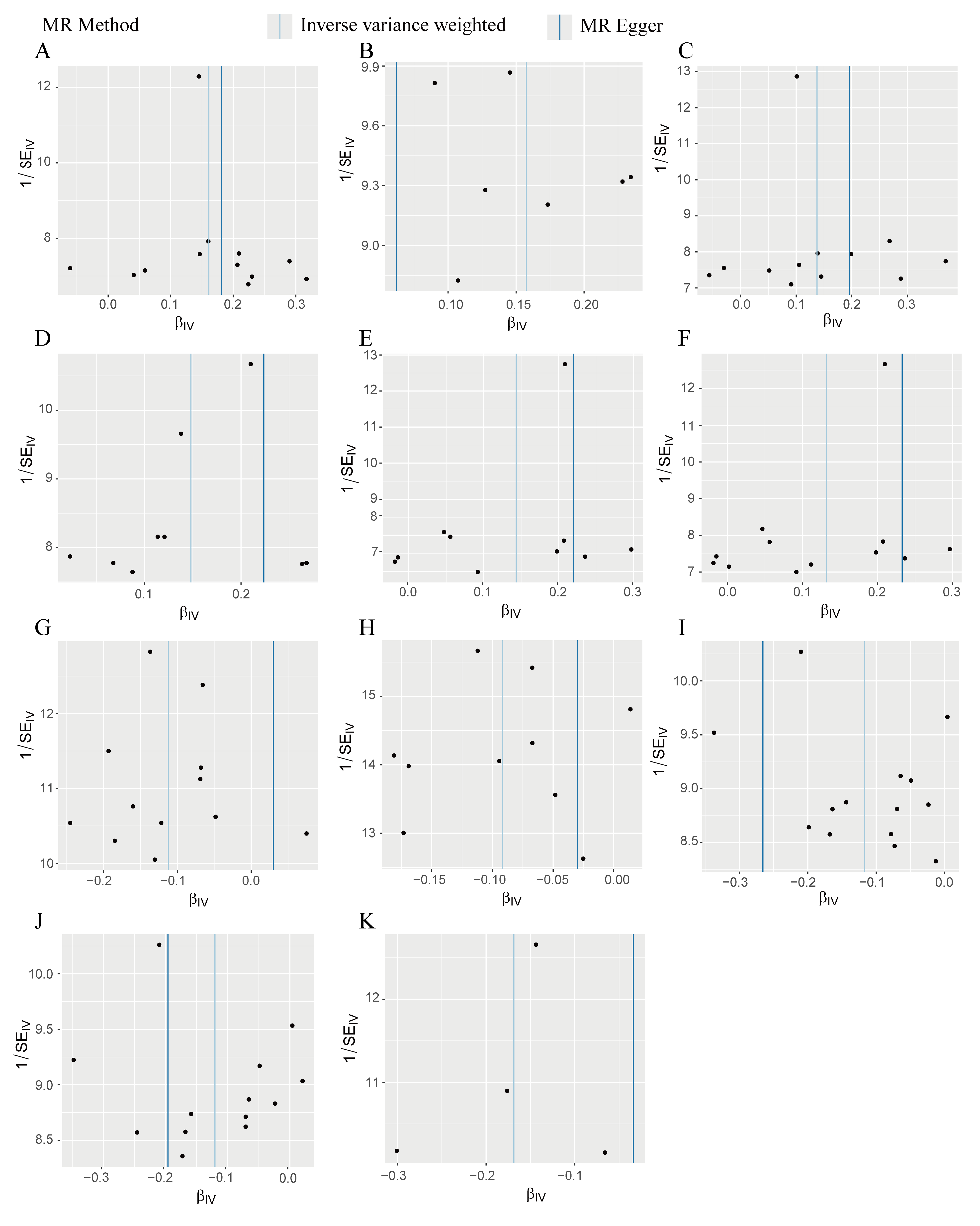


**Figure S2.** Funnel plot of a two-sample Mendelian randomization study of gut microbiota and brain structural connectivity. **(A)** Order *Desulfovibrionales* on Left−hemisphere somatomotor network to right−hemisphere dorsal attention network white−matter structural connectivity. **(B)** Genus *Veillonella* on Left−hemisphere dorsal attention network to right−hemisphere dorsal attention network white−matter structural connectivity. **(C)** Order *Desulfovibrionales* on SNP effect on Left−hemisphere somatomotor network to right−hemisphere somatomotor network white−matter structural connectivity. **(D)** Genus *Escherichia Shigella* on Left−hemisphere dorsal attention network to right−hemisphere limbic network white−matter structural connectivity. **(E)** Family *Desulfovibrionaceae* on Left−hemisphere salience/ventral attention network to right−hemisphere control network white−matter structural connectivity. **(F)** Order *Desulfovibrionales* on Left−hemisphere salience/ventral attention network to right−hemisphere control network white−matter structural connectivity. **(G)** Genus *Ruminococcus gnavus* group on Left−hemisphere limbic network to right−hemisphere default mode network white−matter structural connectivity. **(H)** Genus *Howardella* on Left−hemisphere limbic network to left−hemisphere control network white−matter structural connectivity. **(I)** Family *Rhodospirillaceae* on Left−hemisphere visual network to hippocampus white−matter structural connectivity. **(J)** Order *Rhodospirillales* on Left−hemisphere visual network to hippocampus white−matter structural connectivity. **(K)** Genus *Senegalimassilia* on Right−hemisphere somatomotor network to caudate white−matter structural connectivity.


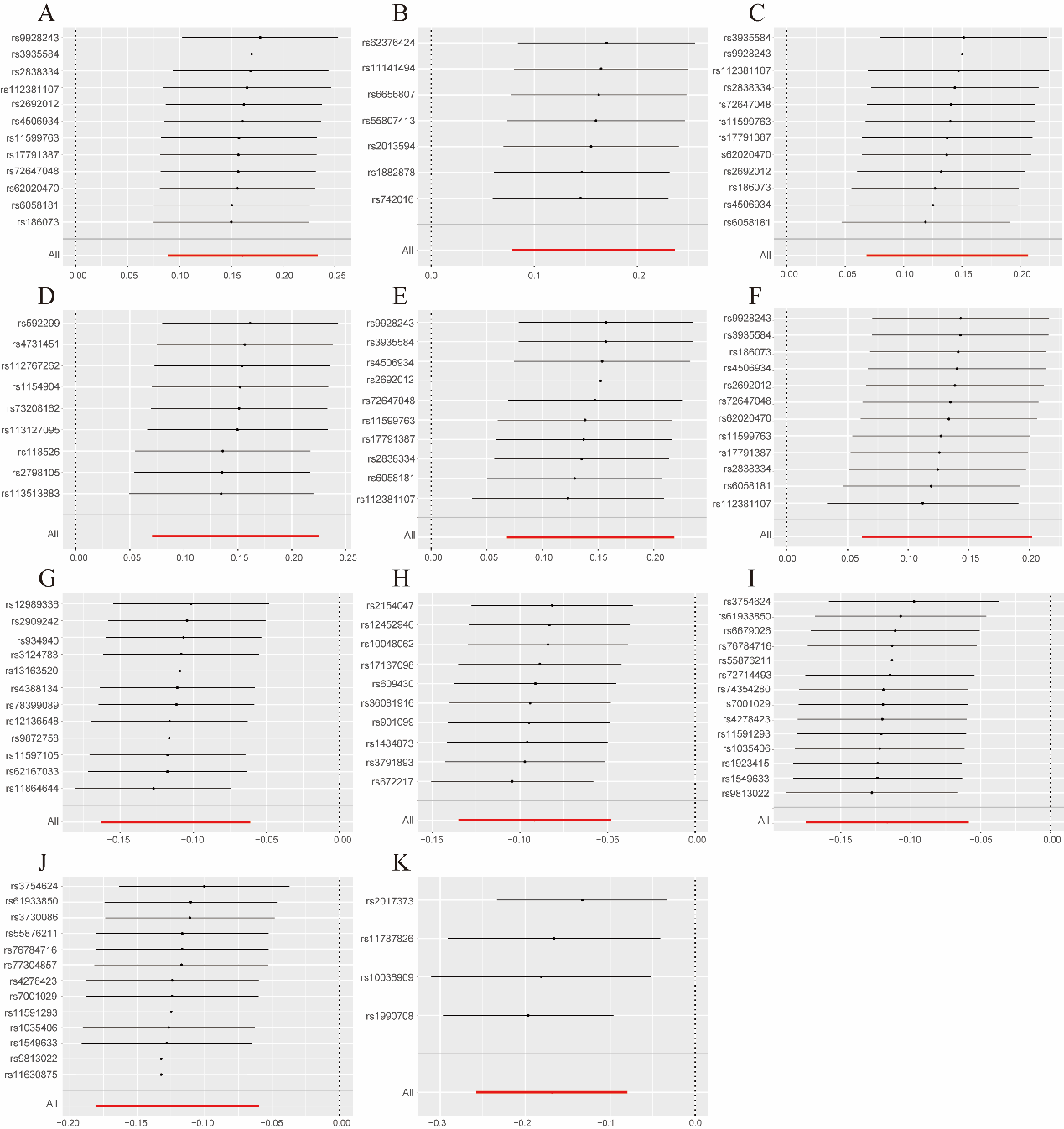


**Figure S3.** Forest plot of a two-sample Mendelian randomization study of gut microbiota and brain structural connectivity. **(A)** Order *Desulfovibrionales* on Left−hemisphere somatomotor network to right−hemisphere dorsal attention network white−matter structural connectivity. **(B)** Genus *Veillonella* on Left−hemisphere dorsal attention network to right−hemisphere dorsal attention network white−matter structural connectivity. **(C)** Order *Desulfovibrionales* on SNP effect on Left−hemisphere somatomotor network to right−hemisphere somatomotor network white−matter structural connectivity. **(D)** Genus *Escherichia Shigella* on Left−hemisphere dorsal attention network to right−hemisphere limbic network white−matter structural connectivity. **(E)** Family *Desulfovibrionaceae* on Left−hemisphere salience/ventral attention network to right−hemisphere control network white−matter structural connectivity. **(F)** Order *Desulfovibrionales* on Left−hemisphere salience/ventral attention network to right−hemisphere control network white−matter structural connectivity. **(G)** Genus *Ruminococcus gnavus* group on Left−hemisphere limbic network to right−hemisphere default mode network white−matter structural connectivity. **(H)** Genus *Howardella* on Left−hemisphere limbic network to left−hemisphere control network white−matter structural connectivity. **(I)** Family *Rhodospirillaceae* on Left−hemisphere visual network to hippocampus white−matter structural connectivity. **(J)** Order *Rhodospirillales* on Left−hemisphere visual network to hippocampus white−matter structural connectivity. **(K)** Genus *Senegalimassilia* on Right−hemisphere somatomotor network to caudate white−matter structural connectivity.
